# Supplementary material for: Learning curve and surgical outcome of robotic assisted colorectal surgery with ERAS program
Source: Sci Rep. 2022 Nov 29;12:20566. doi: 10.1038/s41598-022-24665-w (PMC9709162; doi:10.1038/s41598-022-24665-w)
Supplement: Supplementary file 2 — Supplementary Information 2. [file 41598_2022_24665_MOESM2_ESM.zip › RAL Raw data-Ñ[▒K/053/OP N_ñw╝╨░O▒Kñσ.pdf]

W52 / W52

臺中榮民總醫院

手術室護理記錄

[redacted]

基本資料

- 室別： 開刀房-17室，手術科別： CRS，手術類別： 常規，日期： 2021/01/18
- 進入時間： 1710 ； 麻醉開始時間： 1725 ； Time Out時間： 1848 ； 劃刀時間： 1850 ； 手術結束時間： 0215 送出時間： 0230 ；
- 麻醉方式： GE

  診斷：MUCOAL MELANOMA OF RECTUM

  手術名稱： 1. Robotic (XI) assisted abdominal-perineal resection with end S-colostomy
  2. Intraoperative colonoscopy : (-).
  3. High ligation (+) : At IMA root. Left colic artery and IMV were also ligated via robotic endoclips.
  4. Take down from the splenic flexure : (-)
  5. Robotic endoGIA (45-4.3) X1 was applied to resected the S-colon.
  6. Proximal cut end: 8 cm
  7. Two 12 Fr. penrose drainage tube were placed from the perineal wound into the pelvic cavity.
  8. The perineal wound was closed with interrupted 2-0 Nylon and then covered with gauze.

  手術局部用藥：nil

  術前預防性抗生素：
  - 抗生素名稱一： Veterin 1GM IVD由麻醉護理人員給予
  - 時間： 1830
  - 上次簽章: 紀玉娟

  ，追加預防性抗生素：
  - 抗生素名稱一： Veterin 1GM IVD由麻醉護理人員給予
  - 時間： 2230
  - 上次簽章: 陳心慈
- 手術醫師： 蔣鋒帆，林陳彥，陳映菁，陳凱祥
- 麻醉醫師： VS 蔡志忠
-
  - 刷手護士A ： 廖子晴，時間： 1710，接替刷手一： 邱任宏，時間： 1800，接替刷手二： 蘇郁安，時間： 2200
  - 流動護士A ： 紀玉娟，時間： 1710，接替流動一： 陳心慈，時間： 2200
- 更多接替及流動護士： 接替刷手三： 陳祐溥，時間： 2300，接替流動三： 顏育銹，時間： 0100

皮膚狀況

A-V Shunt： 無

術前： 完整

記錄者： 上次簽章: 紀玉娟

術後： 完整

記錄者： 上次簽章: 顏育？

手術準備

手術臥式 單一手術臥式

-
  - 手術臥式： 仰臥
  - 使用附件： 溫毯 38 ℃，頭圈，腳架，其它： 側身架2組+小Sillicon pad 2個
  - 皮膚準備： 10%Aq Betadine，Hibiscrub，其它： 2%克菌寧
  - 電燒： 使用電燒: 電極片位置： 右大腿，雙極電燒，記錄者： 上次簽章: 紀玉娟

止血帶T： 無

植入物

記錄者： 上次簽章: 顏育？

標本送檢

- 細菌培養 無
- 細胞學 無

- 冰凍切片 無
- 病理標本
  組織名稱一： para-aortic LN
  ,
  組織名稱二： rectum
  ,
  組織名稱三： IMA Root
  , 更多組織名稱
    - 組織名稱四： peritoneum
- 截肢 無

標本交付 標本： 2 袋 ， 標本： 0 盒 ， 標本： 2 瓶 ， 細胞學： 0 管 ， Culture Tube： 0 管 ， 被交付醫師： CR 林陳彥 ，

- 記錄者： 上次簽章: 顏育？
- 時間： 0230

傷口術後敷料

傷口敷料： 傷口敷料： 無

- 請於圖上標示部位：

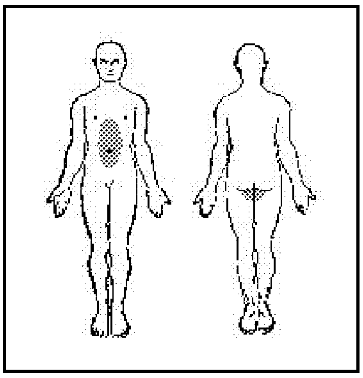

- 傷口敷料： 軟式敷料

導管

導管	天數	導管資訊	插入日期	插入時間	更換日期	拔除日期	拔除時間	拔除原因	預計拔除/更換	預計拔除/更換日期
Foley(1)	3	號碼:16 Fr.; 部位:膀胱;	2021/01/18	1755						
Double Lumen CVC(1)	3	號碼:8 Fr; 深度:15cm; 部位:jugular (右); 象限:RU;	2021/01/18	1740						
Penrose Drain(1)	2	號碼:12 mm.; 部位:臀部;	2021/01/19	0010						
Penrose Drain(1)	2	號碼:12 mm.; 部位:臀部;	2021/01/19	0010						

用物計數

- 手術中出血量： 300 c.c.
- 手術中尿量： 450 c.c.
-
    - 其它： 無

用物計數： 多切口同時縫合

- 1.器械： 正確 ，2.縫針： 正確 ，3.刀片： 正確 ，4.紗布： 正確 ，5.腹部墊： 未使用 ，6.紗球： 未使用 ，7.PEANUTS ： 未使用 ，8.紗條： 未使用 ，9.綵帶： 未使用 ，10.針頭： 未使用 ，11.Vessel loop： 未使用 ，12.Boots： 未使用 ，13.Jelco： 未使用 ，14.棉條： 未使用 ，
    - 第一次：
        - 刷手護士 ： 陳祐嫥
        - 流動護士： 上次簽章: 顏育？
        - 時間： 0030

  ,
    - 第二次：
        - 刷手護士 ： 陳祐嫥

    - 流動護士： 上次簽章: 顏育？
    - 時間： 0045

,

- 第三次：
    - 刷手護士 ： 陳祐婷
    - 流動護士： 上次簽章: 顏育？
    - 時間： 0100

備註

備註： 腹腔鏡scope NO.1 ,Xi Davinci 30度 scope NO.1

- 記錄者： 上次簽章: 顏育？
- 時間： 0230

填寫護理人員_顏育？________

正式摘要

W52 / W52

doctorRank:B
